# Transient Simulation for the Thermal Design Optimization of Pulse Operated AlGaN/GaN HEMTs

**DOI:** 10.3390/mi11010076

**Published:** 2020-01-09

**Authors:** Huaixin Guo, Tangsheng Chen, Shang Shi

**Affiliations:** Science and Technology on Monolithic Integrated Circuits and Modules Laboratory, Nanjing Electronic Devices Institute, Nanjing 210016, China

**Keywords:** AlGaN/GaN HEMTs, thermal simulation, transient channel temperature, pulse width, gate structures

## Abstract

The thermal management and channel temperature evaluation of GaN power amplifiers are indispensable issues in engineering field. The transient thermal characteristics of pulse operated AlGaN/GaN high electron mobility transistors (HEMT) used in high power amplifiers are systematically investigated by using three-dimensional simulation with the finite element method. To improve the calculation accuracy, the nonlinear thermal conductivities and near-junction region of GaN chip are considered and treated appropriately in our numerical analysis. The periodic transient pulses temperature and temperature distribution are analyzed to estimate thermal response when GaN amplifiers are operating in pulsed mode with kilowatt-level power, and the relationships between channel temperatures and pulse width, gate structures, and power density of GaN device are analyzed. Results indicate that the maximal channel temperature and thermal impedance of device are considerably influenced by pulse width and power density effects, but the changes of gate fingers and gate width have no effect on channel temperature when the total gate width and active area are kept constant. Finally, the transient thermal response of GaN amplifier is measured using IR thermal photogrammetry, and the correctness and validation of the simulation model is verified. The study of transient simulation is demonstrated necessary for optimal designs of pulse-operated AlGaN/GaN HEMTs.

## 1. Introduction

AlGaN/GaN high electron mobility transistors (HEMTs) have recently been researched intensively and are considered prospective for high-power RF applications, owing to the advantages such as wide bandgap, high breakdown voltage, and high electron mobility [1,2,3,4]. However, high power applications require high power densities in the active region of GaN devices, which leads to highly localized Joule self-heating and potentially high peak temperatures. The localized self-heating of two dimensional electron gas in the conducting channel limits the highest output power density and decreases its reliability. Therefore, the self-heating effect is a main factor that limits the power density of GaN HEMTs [3,4,5,6]. To exploit the full potential of GaN devices, especially high-power amplifiers, a great concern is the thermal management both from a performance point of view and more importantly to ensure adequate device reliability [2,3,4,5,6,7,8].

For the high-power amplifiers used in radar and communication system, the GaN HEMTs are often operated in pulsed mode, although performance is typically pulse width for the purpose of thermal management, and to maintain the operating channel temperature within a safe limit to avoid thermally activated degradation of the device performance. Meanwhile, the relation between typically pulse width and channel temperature is influenced by thermal design of GaN HEMTs, including the structure of gates and the power density. Previous works on thermal management in GaN HEMTs has been made, but those researches were focused on self-heating effect with different substrate materials, near-junction region thicknesses, and interfacial layers. However, thorough understanding of the effects of pulse width, gate structures, and power density on channel temperatures have not been well addressed, which are desirable for optimal implementation of GaN HEMT used in high-power amplifiers [2,4,7,8,9,10,11]. Meanwhile, these effects can hardly be predicted by measurements because of the limits of the spatial and temporal resolution. Therefore an accurate transient thermal analysis method is highly desired.

In this paper, we analyze the transient thermal characteristics of pulse-operated AlGaN/GaN HEMT used in high power amplifiers. The relationships between channel temperatures and pulse width, gate structures, and power density of GaN amplifiers with kilowatt-level power are analyzed using the finite element method implemented by the commercial simulation software (COMSOL). The simulation details such as the geometry of the multi fingers GaN HEMTs and material properties are presented in Section 2. The numerical results and discussion in Section 3 focus on illustrating the heat spreading effect and optimizing thermal design. Finally, the experimental test for the thermal design of GaN amplifier is shown in Section 4, and some conclusions are drawn in Section 5.

## 2. Device Details and Simulation Methods

**Figure 1:**
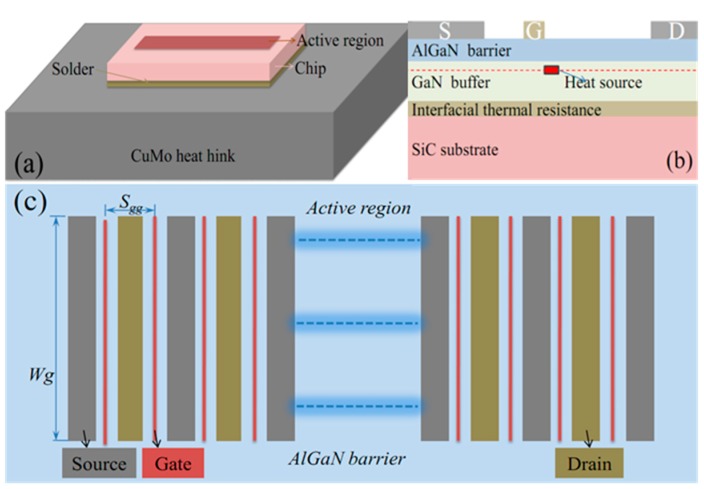
(**a**) Schematic diagram of the actual module for GaN amplifiers; (**b**) the cross section of chip; (**c**) the top active region.

The geometry of the GaN HEMT power amplifiers is shown in Figure 1a. The layer structure of GaN chip consists of an AlGaN barrier, a GaN buffer, an interface of a GaN/SiC, and a SiC substrates to improve calculation accuracy (Figure 1b). In addition, the chip is soldered to a CuMo heat sink with an AuSn joint for efficient thermal management. The length and width of heat sink are defined as twice the sizes of the chip, in order to avoid the effect of thermal simulation model of GaN HEMTs, which is affected by the large size ratio of chip and heat sink, and thus that the calculation accuracy is not affected when the length and width of heatsink are two times the size of chip in simulation model [12]. In order to estimate the relationships between channel temperatures and pulse width, gate structures, and power density of GaN amplifiers, the geometric and operating parameters are designed as Table 1. The active area is defined as the heat sources region of all gates determined by gate pitch spacing and total gate width (Figure 1c). The power density is an important index for the pulse operated device in product application, and it is determined by dividing the total power by total gate width, the total power defined as thermal dissipated power divided by power efficiency. The pulse period is the product of pulse width and its duty cycle. All the variables are shown in Table 1.

The calculations are carried out by the three-dimensional finite element method with COMSOL multiphysics. Here, to improve the calculation accuracy, the nonlinear thermal conductivities of materials, operating conditions, and multilayer physical structures of GaN chip are considered and treated appropriately in our numerical analysis. For the nonlinear thermal conductivities of materials, temperature-dependent thermal conductivities of AlGaN, GaN, and SiC materials of near-junction region of GaN chip have been introduced into the model by employing Kirchhoff’s transformation, with these thermal parameters of the device shown in Table 2 [3,12,13,14,15]. Operating conditions such as the thermal accumulation, the environmental issues around the GaN amplifier, and heat transfer of the CuMo heat hink are considered, the themo-electro effect is considered as the source of thermal accumulation. The natural convection is applied on the external surfaces of GaN amplifier, and the bottom of the heat sink is set as an isothermal surface plane with constant temperature of 333.15 K (ambient temperature). Meanwhile, this model takes into account the multilayer physical structure factors of the near-junction region, including details of AlGaN barrier, GaN buffer, interfacial layer of GaN/SiC and SiC substrate layers. However, it is challenging to introduce all these parameters simultaneously into the simulation model since it might cause some problems such as the size effect, huge amounts of simulation grid, and failure of convergence, especially for transient simulation.

In this paper, some theoretical hypotheses are applied to solve the confliction relation between calculating precision and feasibility under transient simulation for pulse-operated AlGaN/GaN HEMT used in kilowatt-level power amplifiers. First, to avoid huge amounts of simulation grid and achieve feasibility of three-dimensional calculation, the themo-electro effect of GaN amplifier was simplified into heat sources of which the cross section of the model is illustrated in Figure 1b, and the heat sources represent the constant heat flux generated by dissipated power directly under the gates, and the length and width of cross section of heat sources is designed as 0.5 μm and 0.1 μm based on the size of gates of GaN chip (the length of gates is 0.25 μm). Second, to solve the size effect between the chip and the packaging, the gate/drain/source multi-layer metallization was omitted because of small-structural complexity effect [3,4,16]. And the AlGaN barrier material with the 20 nm thickness was supposed to a thin layer, the thin layer had only heat transfer characteristics. Meanwhile, the interface of GaN/SiC is a thick AlN nucleation layer with 20 nm thickness that involves intricate resistance mechanisms, including defects, dislocations, and interfacial disorders, these mechanisms seriously damage the thermal property, therefore heat spreading capacity of this interface of GaN/SiC was represented as a single effective interface thermal resistance in our model. Finally, to reduce the total computing time, only a quarter of device was simulated, because of their structural symmetry [3,12,13,14,15,16,17,18,19,20,21].

## 3. Simulation Results and Discussion

### 3.1. Transient Channel Temperature in Pulsed Operation

**Figure 2:**
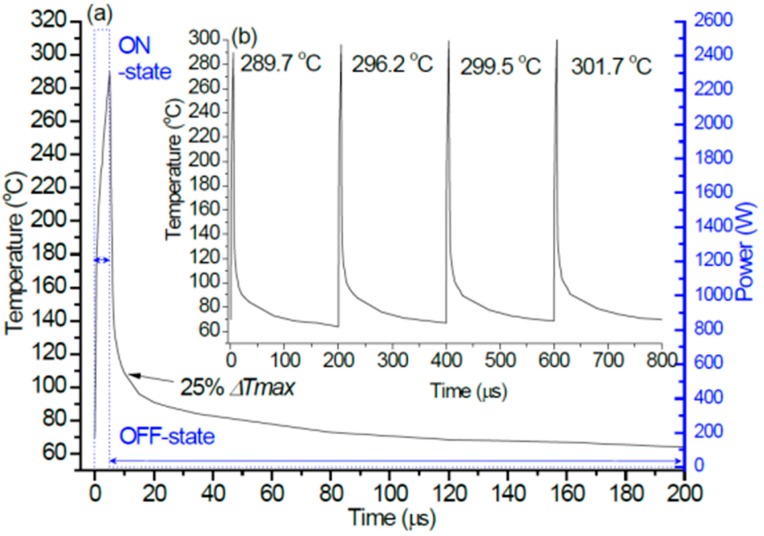
(**a**) Transient channel temperature with pulsed-mode power during one pulse period; (**b**) periodic transient pulses temperature

The analyses of transient channel temperature were carried out by the power density of 26.56 W/mm within four pulse repetition periods. A pulse width of 5 μs was used with the pulse period of 200 μs, and the values of gate pitch spacing, gate width and total gate width are 11.5 μm, 342.86 μm, and 96 mm, respectively. The first observation in Figure 2a is that the thermal response changes rapidly with the sudden power rise because of the Joule heating, the trend is that the channel temperature rises instantly at the start, and then continues to rise in approximate linearity with the increase of load power time throughout the ON-state portion. At the OFF-state, the channel temperature reduces immediately as power returns to 0 W, which drops to 25% temperature increment (Δ*T*_max_ = maximal channel temperature—ambient temperature) when the time was 8.5 μs, then the channel temperature drops slowly until the next ON-state.

Meanwhile, as the inset Figure 2b presents, the thermal response is same in different pulse repetition periods, but the channel temperature is increased by the rise of pulse repetition period, particularly the maximal channel temperature is at the end of the pulse (ON-state) because of heat accumulating effect. The maximal channel temperatures are 289.7 °C, 296.2 °C, 299.5 °C, and 301.7 °C with the rise of pulse repetition period, respectively. The explanation is that thermophysical properties of chip materials will reduce because of the nonlinear thermal conductivities of materials, and this leads to the increase of heat accumulation with the rise of pulse repetition period. Meanwhile, the increment of maximal channel temperature is reduced from 6.5 K to 2.2 K, meaning that heat accumulating effect will gently reach saturation in the next several or longer pulse repetition periods, and the channel temperature will be balanced.

**Figure 3:**
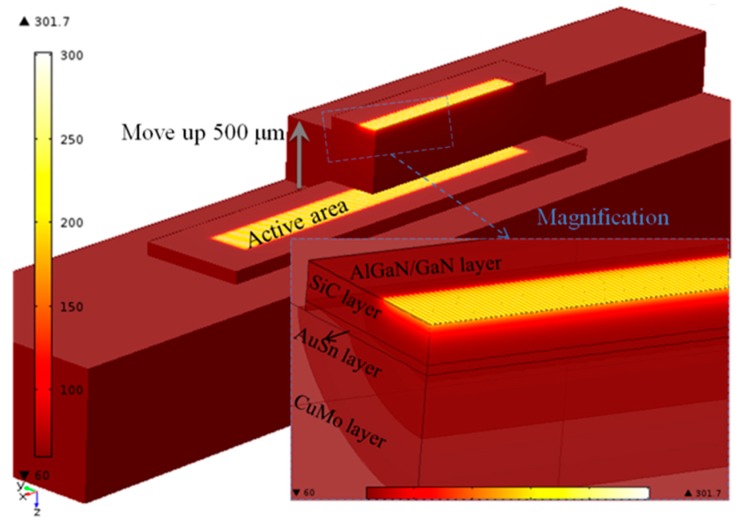
Temperature distribution and isothermal surfaces of device.

In addition, the temperature distribution under the time as 605 μs is shown in Figure 3, and the result shows that the heat mainly focuses on the region of active area. The paths of heat transfer are directly reflected in isothermal surfaces as shown in Figure 3 (magnification), and most of the heat is directly spread into SiC substrate through GaN buffer layer, then continued to transfer downward, and those heat ultimately gets extracted by the CuMo heat hink through the AuSn joint layer.

### 3.2. The Pulse Width Effect

**Figure 4:**
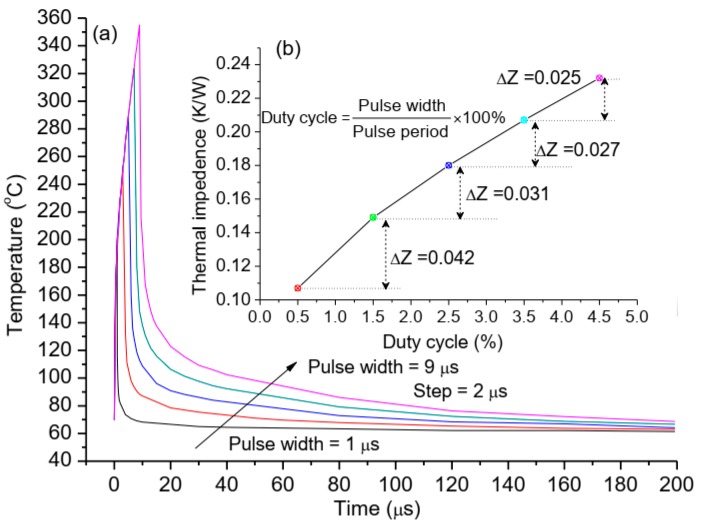
(**a**) Dependence of transient channel temperature on pulse width during one pulse period; (**b**) the impact of duty cycle on thermal impedance of device; the pulse width is 1 μs, 3 μs, 5 μs, 7 μs, and 9 μs, respectively, according to the duty cycle of 0.5%, 1.5%, 2.5%, 3.5%, and 4.5%.

Thermal investigation on pulse width was carried out by varying the pulse width from 1 μs to 9 μs at the pulse period of 200 μs, these values are based on the performance indicators of our GaN HEMT power amplifier in the practical application. The values of gate pitch spacing, gate width, and total gate width are 11.5 μm, 342.86 μm, and 96 mm, respectively, and the power density is 26.56 W/mm. The results indicate the channel temperatures are seriously affected by pulse width as shown in Figure 4a. Especially in the ON-state, it means load pulsed power time shown in Figure 2a, corresponding to 1 μs, 3 μs, 5 μs, 7 μs, and 9 μs respectively in Figure 4a, the trends of the channel temperatures of different pulse width have been essentially same in the load power time, and the channel temperature has been normalized to the maximal temperature increase at the end of the respective pulse, this means that the greater the pulse width the higher the maximal channel temperature. At the OFF-state, the channel temperatures reduce immediately, and the times (*t*_0.25_) when the channel temperature drops to 25% temperature increment are 1.4 μs, 4.8 μs, 8.5 μs, 12 μs, and 15.5 μs, respectively. The cooling ratio of *t*_0.25_ divided by the pulse width are 1.4, 1.6, 1.7, 1.7, and 1.7, respectively. This demonstrates that there exists a critical saturation for the cooling ratio with the increasing of the pulse width. It is noteworthy that the thermal impedance of device increases with the rise of pulse width shown in the inset Figure 4b, the relation between pulse width and duty cycle is also decided by the formula in the inset Figure 4b, and we find that this increase (ΔZ) of thermal impedance becomes smaller and presents nearly a linear approximation of the exponential function when the duty cycle is more than 2.5%. This suggests that the thermal impedance of device is relatively high sensitivity to duty cycle.

**Figure 5:**
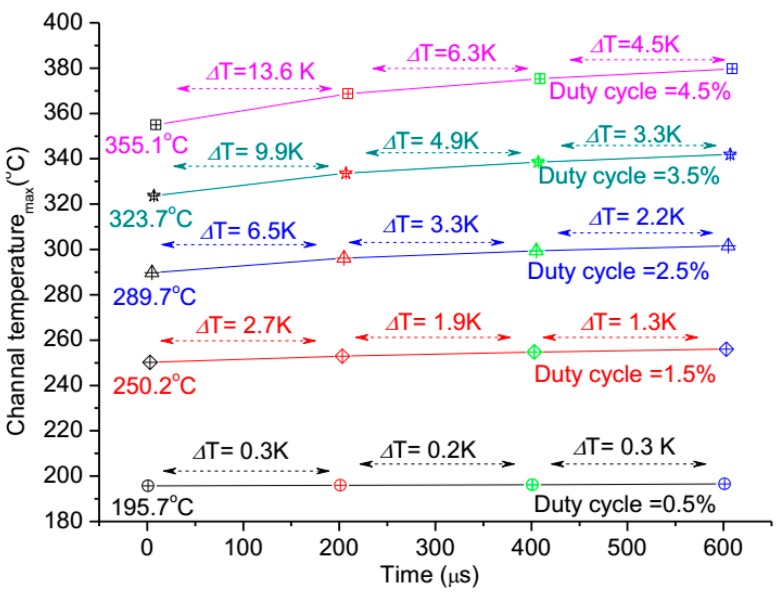
The impact of pulse width on maximal channel temperature during four pulse repetition periods.

Meanwhile, the heat accumulating effect on the pulse width is analyzed, as shown in Figure 5, within four pulse repetition periods. The maximal channel temperatures were calculated in four pulse repetition periods with different duty cycles. Results indicate that there is almost no change (Δ*T*) of the maximal channel temperature in four pulse repetition periods when the duty cycle is 0.5%, but the change (Δ*T*) goes up sharply with the duty cycle increase, and the trend of the Δ*T* becomes small gradually as the pulse period going on. This demonstrates that the duty cycle has a larger impact on the heat accumulating effect for the device in pulsed operation, but an optimal duty cycle should exist for the contradiction between large duty cycle applications and little heat accumulating effect.

### 3.3. The Gate Structures Effect

**Figure 6:**
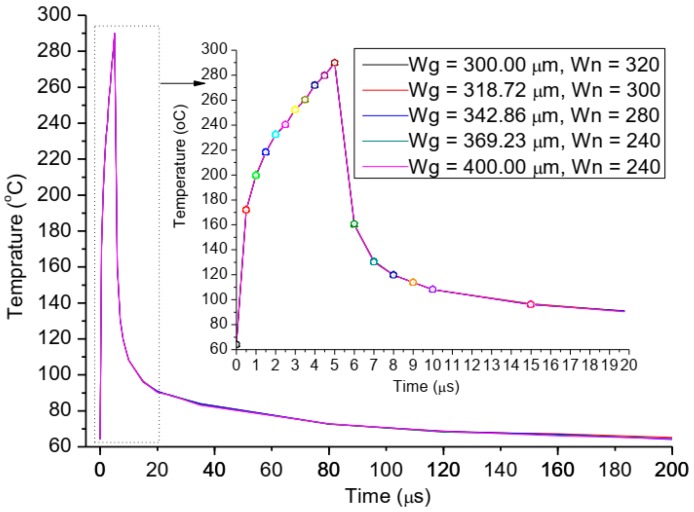
The relationships between transient channel temperature and the changes of the gate width and gate fingers, Wg is the width of a single gate, and Wn is the numbers of gate fingers.

To consider the gate structures (the changes of gate width and gate fingers) effect, we keep the power density and pulse width as 26.56 W/mm and 5 µs respectively, meaning that the total gate width and gate pitch spacing is also a constant value which is 96 mm and 10 μm, respectively, and this represents that the length and width of active region is controlled when the active area of heat sources region is fixed. The thermal investigation on gate structures effect was carried out by varying the gate width (*W*g) from 400 to 300 μm (Figure 6), and according to 240, 260, 280, 300, and 320 gate fingers (*W*n), respectively. The results of transient simulation are shown Figure 6 in one pulse period (200 μs). We find that the trends of channel temperatures for all five gate structures are the same, and the channel temperature variations are the same in the over pulse period. This result indicates the changes of gate fingers and gate width have no effect on the channel temperature of the device when the total gate width and active area is also a constant value, significantly, meaning that the surface profile of active area are free to control for thermal design of GaN HEMT device. This is very important for electric and thermal collaborative design of high power GaN device, and provides more space for electric design.

### 3.4. The Power Density Effect

**Figure 7:**
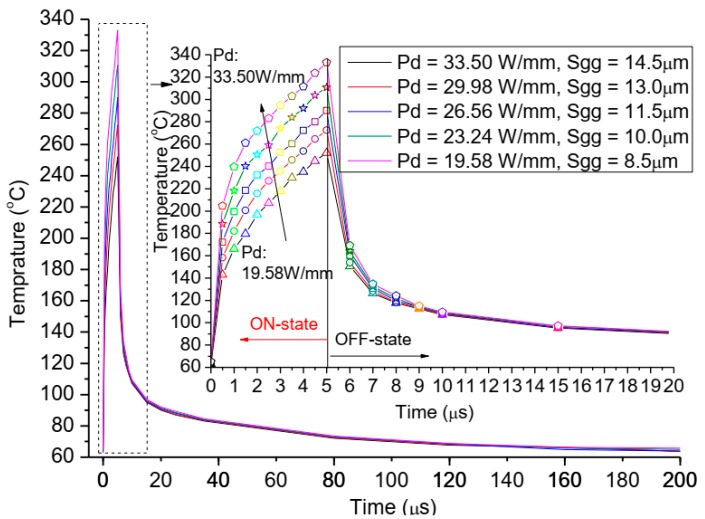
The relationships between transient channel temperature and the changes of power density and gate pitch spacing.

The change of power density means that the total gate width will change, because that they are interrelated when the total power and the active area of heat sources region are kept fixed at predetermined value in Table 1. The power densities are designed as 33.50 W/mm, 29.98 W/mm, 26.56 W/mm, 23.24 W/mm, and 19.58 W/mm, respectively, which correspond to the values of total gate width as 76.115 mm, 85.029 mm, 96.001 mm, 109.715 mm, 130.287 mm, respectively, and the gate pitch spacing (*Sgg*) are about 14.5 μm, 13 μm, 11.5 μm, 10 μm, and 8.5 μm, respectively, the gate width is fixed as 342.86 μm. The results, as shown in Figure 7, present that the trends of the influence on the channel temperature by the power density (*Pd*) are basically similar in one pulse period, but the degree of the influence increases greatly with the rise of power density throughout the ON-state portion. At the OFF-state, the channel temperatures reduce immediately in 0.5 μs as power returns to 0 W, and the values of channel temperatures in different power density are near when the time is more than 9 μs, but still the more the power density, higher the channel temperature.

**Figure 8:**
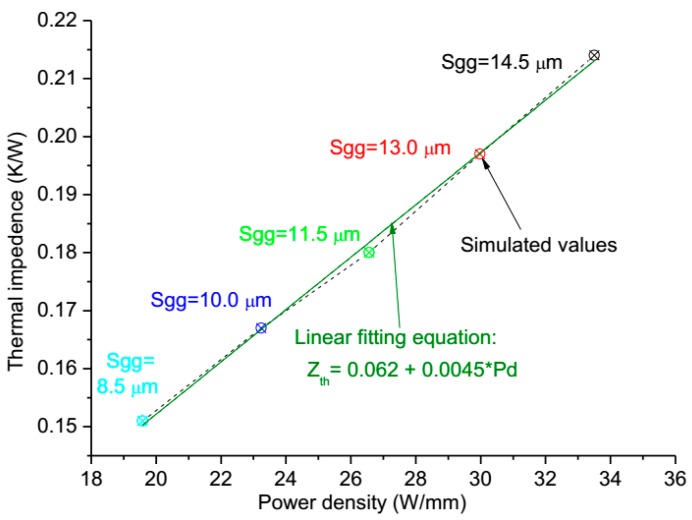
The impact of power density on thermal impedance of device.

Besides, the thermal impedance of device is analyzed, as shown in Figure 8, and we observe that the thermal impedance (*Z_th_*) is a linear approximation of the exponential function with respect to the power density and the gate pitch spacing, and the linear fitting equation is: *Z_th_* = 0.062 + 0.0045 × *P*_d_. Results demonstrate that power density can extremely affect the channel temperature of the device when the total power, active area, and gate width are also constant values, meaning that we can reduce the channel temperature by the combined contribution of decreasing the power density and gate pitch spacing for the thermal design of GaN HEMT device.

## 4. Experimental Test

**Figure 9:**
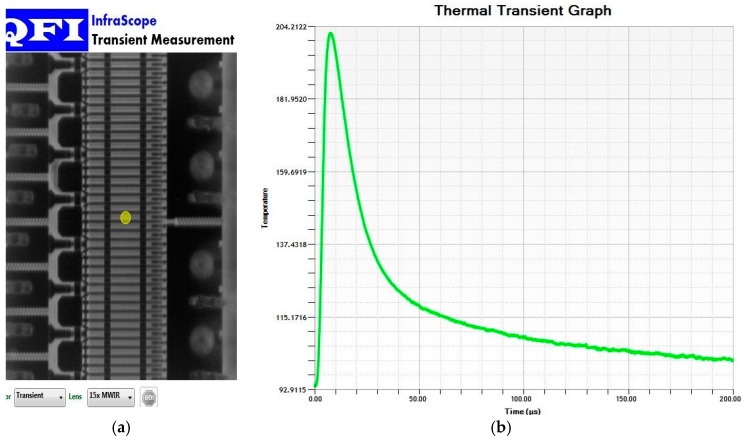
(**a**) The test position of GaN amplifier; (**b**) the transient thermal response of the GaN amplifier under 5 μs pulse width measured by IR thermal photogrammetry.

A GaN amplifier with kilowatt-level power has been designed based on the above thermal analysis, the power density and gate pitch spacing are designed as 19.58 W/mm and 8.5 μm in order to gain low channel temperature, when total power and active area of heat sources region are kept fixed at a predetermined value, as shown in Table 1. The value of gate width of single gate and total gate width is and 342.86 μm 130.287 mm, respectively. We have the IR thermal photogrammetry, and use the 15× infrared objective for high spatial resolution. The channel temperature of the GaN amplifier operated with a pulse width of 5 μs had been measured by the initial model of the infrared microscope. The transient thermal response and the test position of the GaN amplifier is shown in Figure 9, and the simulation of the GaN amplifier is shown in Figure 7. The experiment result shows that the maximal channel temperature is 202.19 °C, the experimental value is below the simulate value (252.11 °C shown in Figure 7), around 80.19%. The deviation between calculated and tested data is about 20%, this is primarily due to the low temporal and spatial resolution, surface temperature of IR thermal photogrammetry, and the correctness and validation of the simulation model is demonstrated. Meanwhile, comparison of transient thermal response of the GaN amplifier under experiment and simulation shows that the trends of channel temperatures are basically similar in one pulse period. But, the experimental value of the maximal channel temperature has shown “style drift,” meaning that the maximal channel temperature is not at the end of the pulse, mainly because of low resolution in time and surface temperature of IR thermal photogrammetry. The accuracy and precision in the microsecond range of temperature measurements is always a difficult subject in this field.

## 5. Conclusions

A theoretical transient thermal model based on the finite element analysis is presented to understand the relationships between the channel temperatures and pulse width, gate structures, and power density of GaN amplifiers operating at kilowatt-level power. Thermal response in pulsed operation indicates that the channel temperature sharply rises and goes up linearly until the end of pulse, while it reduces immediately within the tenth of the pulse width as power returns to 0 W at the OFF-state. In the meantime, the periodic transient pulse temperature and the temperature distribution are shown to be the reason of heat accumulating effect. The simulation results of pulse width effect show that the channel temperature rises with the increase of the duty cycle but in a decreasing trend, and there is an optimal duty cycle for heat accumulating effect in stable period pulses. Meanwhile, the power density shows certain influence on channel temperature, hence we can reduce the maximal channel temperature by the combined contribution of decreasing the power density and gate pitch spacing for the thermal design. Furthermore, when the total gate width and active area remain constant, note that the changes of gate fingers and gate width have no effect on the channel temperature when the total gate width and active area show constant values, therefore, the surface profile of active area are free to control for thermal design of GaN HEMT device. Finally, the correctness and validation of the simulation model are demonstrated by thermal test of IR photogrammetry.

## Figures and Tables

**Table 1:** Geometric and working parameters of the designed device.

Definition	Value	Definition	Value
Length of chip	4000 μm	AlGaN barrier thickness	20 nm
Width of chip	860 μm	GaN buffer thickness	2.0 μm
Length of heat hink	8000 μm	SiC substrate thickness	80 μm
Width of heat hink	1720μm	Solder thickness	20 μm
Heat sink thickness	1000 μm	Pulse period	200 μs
Power efficiency	50%	Pulse width	Variables
Total power	2550 W	Power density, and gate pitch spacing	Variables
Active area of heat sources region	1.104 mm^2^	Gate fingers, and gate width	Variables

**Table 2:** Thermal parameters used in the simulation.

Material	Thermal Conductivity (W/m·K)	Material	Thermal Conductivity (W/m·K)
AlGaN	25 × (T/300)^−1.44^	SiC	387 × (T/293)^−1.49^
GaN	150 × (T/300)^−1.42^	CuMo	167
AuSn	57	-	-
Interfacial thermal resistance		10 K·m^2^/GW	

## References

[1] Amano H., Baines Y., Beam E., Borga M., Bouchet T., Chalker P.R., Charles M., Chen K.J., Chowdhury N., Chu R. (2018). The 2018 GaN power electronics roadmap. J. Phys. D Appl. Phys..

[2] Baczkowski L., Jacquet J.C., Jardel O., Gaquière C., Moreau M., Carisetti D., Brunel L., Vouzelaud F., Mancuso Y. (2015). Thermal characterization using optical methods of AlGaN/GaN HEMTs on SiC substrate in RF operating conditions. IEEE Trans. Electron Devices.

[3] Guo H., Kong Y., Chen T. (2017). Thermal simulation of high power GaN-on-diamond substrates for HEMT applications. Diam. Relat. Mater..

[4] Michael F., Patrick M., Avram B. (2016). Modeling thermal microspreading resistance in via arrays. J. Electron. Packag..

[5] Pavlidis G., Pavlidis G., Heller E.R., Moore E.A., Vetury R., Graham S. (2017). Characterization of AlGaN/GaN HEMTs using gate resistance thermometry. IEEE Trans. Electron Devices.

[6] Chou H.P., Cheng S., Cheng C.H., Chuang C.W. Thermal behavior investigation of cascode GaN HEMTs. Proceedings of the 3rd International Conference on Industrial Application Engineering.

[7] Nigam A., Bhat T.N., Rajamani S., Dolmanan S.B., Tripathy S., Kumar M. (2017). Effect of self-heating on electrical characteristics of AlGaN/GaN HEMT on Si (111) substrate. AIP Adv..

[8] Jones J.P., Heller E., Dorsey D., Graham S. (2015). Transient stress characterization of AlGaN/GaN HEMTs due to electrical and thermal effects. Microelectron. Reliab..

[9] Ishizaki T., Yanase M., Kuno A., Satoh T., Usui M., Osawa F., Yamada Y. (2015). Thermal simulation of joints with high thermal conductivities for power electronic devices. Microelectron. Reliab..

[10] Asubar J.T., Yatabe Z., Hashizume T. (2014). Reduced thermal resistance in AlGaN/GaN multi-mesa-channel high electron mobility transistors. Appl. Phys. Lett..

[11] Darwish A., Bayba A.J., Hung H.A. (2015). Channel temperature analysis of GaN HEMTs with nonlinear thermal conductivity. IEEE Trans. Electron Devices.

[12] Schwitter B.K., Parker A.E., Mahon S.J., Fattorini A.P., Heimlich M.C. (2014). Impact of bias and device structure on gate junction temperature in AlGaN/GaN-on-Si HEMTs. IEEE Trans. Electron Devices.

[13] Bertoluzza F., Delmonte N., Menozzi R. (2009). Three-dimensional finite element thermal simulation of GaN-based HEMTs. Microelectron. Reliab..

[14] Chen X.P., Donmezer F.N., Kumar S., Graham S. (2014). A numerical study on comparing the active and passive cooling of AlGaN/GaN HEMTs. IEEE Trans. Electron Devices.

[15] Wang A., Tadjer M.J., Anderson T.J., Baranyai R., Pomeroy J.W., Feygelson T.I., Hobart K.D., Pate B.B., Calle F. (2013). Impact of intrinsic stress in diamond capping layers on the electrical behavior of AlGaN/GaN HEMTs. IEEE Trans. Electron Devices.

[16] Guo H., Han P., Chen T. (2017). Study of thermal simulation technology for GaN power device. Res. Prog. SSE.

[17] Agarwal G., Kazior T., Kenny T., Weinstein D. (2017). Modeling and analysis for thermal management in gallium nitride HEMTs using microfluidic cooling. J. Electron. Packag..

[18] Heller E., Crespo A. (2013). Electro-thermal modeling of multifinger AlGaN/GaN HEMT device operation including thermal substrate effects. Microelectron. Reliab..

[19] Donmezer N., Islam M., Yoder P.D. (2015). The impact of nongray thermal transport on the temperature of AlGaN/GaN HFETs. IEEE Trans. Electron Devices.

[20] García S., Torre I., Mateos J., González T., Pérez S. (2016). Impact of substrate and thermal boundary resistance on the performance of AlGaN/GaN HEMTs analyzed by means of electro-thermal Monte Carlo simulations. Semicond. Sci. Technol..

[21] Denu G.A., Mirani J.H., Fu J. (2017). FEM thermal analysis of Cu/diamond/Cu and diamond/SiC heat spreaders. AIP Adv..

